# Coenzyme Q10 and Neurological Diseases

**DOI:** 10.3390/ph2030134

**Published:** 2009-12-01

**Authors:** Michelangelo Mancuso, Daniele Orsucci, Valeria Calsolaro, Anna Choub, Gabriele Siciliano

**Affiliations:** Department of Neuroscience, Neurological Clinic, University of Pisa, Tuscany, Italy

**Keywords:** coenzyme Q10, idebenone, mitochondria, mitochondrial diseases, neurodegeneration

## Abstract

Coenzyme Q10 (CoQ10, or ubiquinone) is a small electron carrier of the mitochondrial respiratory chain with antioxidant properties. CoQ10 supplementation has been widely used for mitochondrial disorders. The rationale for using CoQ10 is very powerful when this compound is primary decreased because of defective synthesis. Primary CoQ10 deficiency is a treatable condition, so heightened “clinical awareness” about this diagnosis is essential. CoQ10 and its analogue, idebenone, have also been widely used in the treatment of other neurodegenerative disorders. These compounds could potentially play a therapeutic role in Parkinson’s disease, Huntington’s disease, amyotrophic lateral sclerosis, Friedreich’s ataxia, and other conditions which have been linked to mitochondrial dysfunction. This article reviews the physiological roles of CoQ10, as well as the rationale and the role in clinical practice of CoQ10 supplementation in different neurological diseases, from primary CoQ10 deficiency to neurodegenerative disorders.

## Introduction

**Figure 1:**
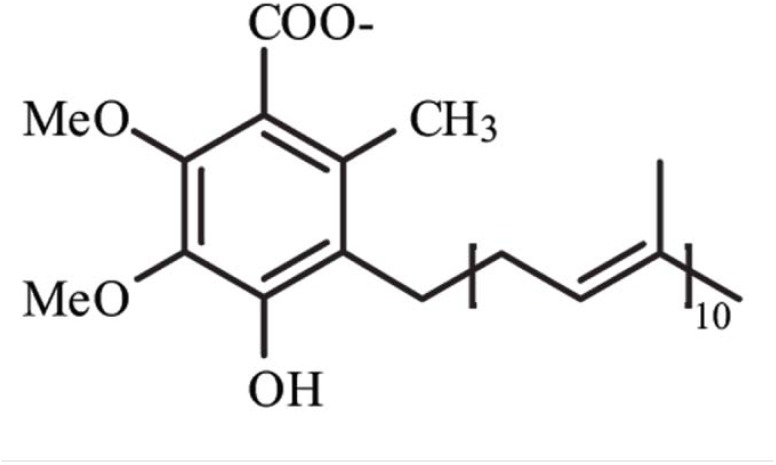
Structure of Coenzyme Q10. Me, methyl groups.

**Figure 2:**
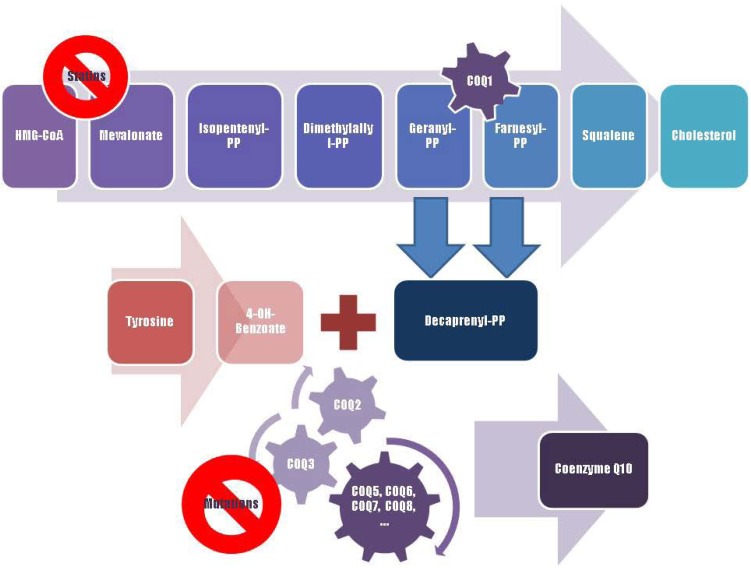
A schematic representation of Coenzyme Q10 biosynthesis. The sequence of cellular reactions that leads to farnesyl-PP is the mevalonate pathway. Farnesyl-PP is the common substrate for the synthesis of cholesterol, dolichol, and Coenzyme Q10, as well as for prenylation of proteins. Coenzyme Q10 contains also a benzoate ring originating from tyrosine. 3-hydroxy-3-methylglutaryl coenzyme A (HMG-CoA) reductase inhibitors, or statins, block production of mevalonate, a critical intermediary in the cholesterol synthesis pathway. A hypothesized mechanism of statin myopathy involve mitochondrial dysfunction caused by reduced intramuscular coenzyme Q10. After 4-OH-benzoate and decaprenyl-PP are produced, at least seven enzymes (encoded by *COQ2-8* genes) catalyze condensation, methylation, decarboxylation, and hydroxylation reactions needed to synthesize Coenzyme Q10. Abnormalities in any part of this metabolic cascade will cause primary CoQ10 deficiency. PP, pyrophosphate.

Coenzyme Q10 (CoQ10), or ubiquinone, is an endogenously synthesized lipid (Figure 1). Intracellular synthesis, which depends on the mevalonate pathway, is the major source of CoQ10 (Figure 2). The mevalonate pathway is a sequence of cellular reactions leading to farnesyl pyrophosphate, the common substrate for the synthesis of cholesterol, dolichol, dolichyl phosphate, CoQ10, and for protein prenylation (a post-translational modification necessary for the targeting and function of many proteins) [1]. Cells synthesize CoQ10 de novo, starting with synthesis of the parahydroxybenzoate ring and the polyisoprenyl tail, which anchors CoQ10 to membranes [1]. The length of this tail varies among different organisms. In humans, the side chain is comprised of ten isoprenyls producing CoQ10 [1] (see Figure 1).

In normal subjects, oral administration of CoQ10 improved subjective fatigue sensation and physical performance during fatigue-inducing workload trials [2]. CoQ10 has been widely used for the treatment of mitochondrial disorders (MD) and other neurodegenerative disorders, as well as its analogue idebenone, which shares an identical modified parahydroxybenzoate ring with CoQ10, but has a short 10-carbon tail. Other potential treatment indications for the use of CoQ10 include migraine [3,4], chronic tinnitus [5], hypertension [6], heart failure and atherosclerosis [7]; however, the role of CoQ10 in such conditions is still an open question. CoQ10, which may ameliorate endothelial function, may be an independent predictor of mortality in chronic heart failure, and there is a rationale for controlled intervention studies with CoQ10 in such condition [8]. Although CoQ10 is also used for the prevention and treatment of cancer, there are no convincing evidences of efficacy [7].

No absolute contraindications are known for CoQ10, and adverse effects are rare [7]. Mild gastrointestinal discomfort is reported in <1% of patients [7]. CoQ10 has an excellent safety record. 

Important pharmacokinetic factors are non-linear absorption and enterohepatic recirculation [9]. Because of its hydrophobicity and large molecular weight, absorption of dietary CoQ10 is slow and limited [10]. In the case of dietary supplements, solubilized CoQ10 formulations show enhanced bioavailability. The T(max) is around 6 h, with an elimination half-life of about 33 hours [10]. The reference intervals for plasma CoQ10 range from 0.40 to 1.91 µmol/L in healthy adults [10]. With CoQ10 supplements there is reasonable correlation between increase in plasma CoQ10 and ingested dose up to a certain point. Animal data show that CoQ10 in large doses is taken up by all tissues including heart and brain mitochondria [10]. This has implications for therapeutic applications in human diseases. Its various formulations demonstrate variation in bioavailability and dosage consistency, and therefore it is important to use brands that have passed independent testing for product purity and consistency [7]. During CoQ10 supplementation plasma CoQ10 levels should be monitored to ensure efficacy, given that there is variable bioavailability between commercial formulations, and known inter-individual variation in CoQ10 absorption [7]. However, plasma levels may not reflect that of the cell and other surrogates such as blood mononuclear cells may be more appropriate [11]. Future CoQ10 research should consider uptake and distribution factors to determine cost-benefit relationships [9]. 

CoQ10 shuttles electrons from complexes I and II and from the oxidation of fatty acids and branched-chain aminoacids (via flavin-linked dehydrogenases) to complex III of the mitochondrial electron transport chain, ETC [1]. Reduced CoQ10 has also antioxidant properties, and therefore may protect membrane lipids, proteins and mitochondrial DNA (mtDNA) against oxidative damage [1]. Mitochondria are dynamic and pleomorphic organelles, which evolved from the aerobic bacteria which about 1.5 billion years ago populated primordial eukaryotic cells, thus endowing the host cells with oxidative metabolism (much more efficient than anaerobic glycolysis) [12]. Mitochondria are composed of a smooth outer membrane surrounding an inner membrane of significantly larger surface area that, in turn, surrounds a protein-rich core, the matrix [12]. They contain two to ten molecules of mtDNA [12]. In humans, the mtDNA is transmitted through maternal lineage [12]. The most crucial task of the mitochondrion is the generation of energy as adenosine triphosphate (ATP), by means of the ETC. The ETC is needed for oxidative phosphorylation (which provides the cell with the most efficient energetic outcome in terms of ATP production), and consists of four multimeric protein complexes located in the inner mitochondrial membrane [12]. The ETC also requires cytochrome *c* (cyt *c*) and CoQ10. Electrons are transported along the complexes to molecular oxygen (O2), finally producing water [12]. At the same time, protons are pumped across the mitochondrial inner membrane, from the matrix to the intermembrane space, by complexes I, III, and IV. This process creates an electrochemical proton gradient. ATP is produced by the influx of protons back through the complex V, or ATP synthase (the “rotary motor”) [12]. This metabolic pathway is under control of both nuclear (nDNA) and mitochondrial genomes [12]. The human mtDNA encodes information for mitochondrial transfer RNAs (tRNAs), for ribosomal RNAs (rRNAs), and for 13 subunits of the ETC [12]. The rest of the mitochondrial proteins are encoded by genes in the nuclear chromosomes, and finally imported into the mitochondrion [12].

Mitochondria also play a central role in apoptotic cell death [13], and mitochondrial dysfunction has been implicated in the pathogenesis of several neurodegenerative diseases, such as amyotrophic lateral sclerosis (ALS), Azheimer’s (AD) and Parkinson’s disease (PD) [13]. Oxidative stress is an earlier event associated with mitochondrial dysfunction [13]. The transport of high-energy electrons through the mitochondrial ETC is a necessary step for ATP production, but it is also source of reactive oxygen species (ROS) production. The sites for ROS production in mitochondrial ETC are normally ascribed to the activity of complexes I and III [13]. On the other hand, the ETC is not universally accepted as the major site for mitochondrial ROS generation. Other mitochondrial components, such as monoamine oxidases and p66(Shc), might contribute to ROS generation [14].

The accumulation of ROS can potentially damage bio-molecules, including lipids, proteins and nucleic acids [13]. The accumulation of nDNA and mtDNA oxidative damage is thought to be deleterious in post-mitotic cells such as neurons, where DNA cannot be replaced through a cellular division mechanism [13]. Indeed, oxidative base modifications to mtDNA could potentially cause bioenergetic dysfunctions resulting in cell death [13]. The cells possess an intricate network of defense mechanisms (mitochondrial manganese superoxide dismutase, glutathione peroxidase and other molecules) to neutralize excessive accumulation of ROS and, under physiological conditions, are able to cope with the flux of ROS [13]. Oxidative stress describes a condition in which cellular antioxidant defences are insufficient to keep the levels of ROS below a toxic threshold [13]. 

The mtDNA is particularly sensitive to oxidative damage because of its proximity to the inner mitochondrial membrane, where oxidants are formed, and because it is not protected by histones and is inefficiently repaired [13]. Because several of the mtDNA genes encode for subunits of the mitochondrial ETC, oxidative mtDNA damage, if not correctly repaired, could result in mutations and deletions disrupting the function of genes involved in the production of ATP, ultimately leading to mitochondrial dysfunction, increased production of ROS, and cellular death [13].

Several studies investigated the role of CoQ10 as a neuroprotective agent *versus* ROS damage and apoptotic cell death. CoQ10 may act by stabilizing the mitochondrial membrane when neuronal cells are subjected to oxidative stress [15]. Pre-treatment with water-soluble CoQ10 maintained mitochondrial membrane potential during oxidative stress and reduced the amount of mitochondrial ROS generation [15]. The evidence of mitochondrial involvement in neurodegenerative diseases allowed the hypothesis that CoQ10 may have a protective role in such diseases [13]. 

For instance, increasing evidence suggests that AD is associated with oxidative damage and mitochondrial dysfunction [13,16]. Exogenous CoQ10 was found to protect neuroblastoma cells from β-amyloid neurotoxic effect; dietary supplementation of CoQ10 to AD mice suppressed brain protein carbonyl levels, which are markers of oxidative damage [13]. This suggests that oral CoQ10 may be a viable antioxidant strategy for neurodegenerative disease [16]. The efficacy of CoQ10 treatment against β-amyloid induced mitochondrial dysfunction has been evaluated also in brains of diabetic rats, where CoQ10 treatment was found to attenuate the decrease in oxidative phosphorylation and avoided the increase in hydrogen peroxide production induced by the neurotoxic peptide [17]. An *in vivo* volume MRI study on mice with mutation in the amyloid precursor protein showed that CoQ10 significantly delayed hemispheric and hippocampal atrophy [18]. Furthermore, the efficacy of CoQ10 as a neuroprotective factor against cognitive impairment has been evaluated in mice with reduced cognitive performance [19]. In aged mice CoQ10, combined with alpha-tocopherol, could have a role in improving learning [20]. 

Moreover, the antioxidant function of CoQ10 has been also studied in noise-induced hearing loss (NIHL). The mitochondrial ETC is source of ROS also in NIHL, and anti-oxidants and free-radicals scavengers have been shown to attenuate the damage [21]. The therapeutic application of CoQ10 is limited by the lack of solubility and poor bioavailability, therefore it is a challenge to improve its water solubility in order to ameliorate the efficacy in tissues and fluids [21]. Fetoni and co-workers [21] reported that the administration of a water-soluble CoQ10 formulation to a guinea pig model of acoustic trauma resulted to prevent apoptosis and improved hearing. The effectiveness of CoQ10 was compared with a soluble formulation of CoQ10 (multicomposite CoQ10 Terclatrate, Q-ter) [21]. Functional and morphological studies were carried out, and animals injected with Q-ter showed a greater degree of activity in preventing apoptosis and in improving hearing [21]. These data confirm that solubility of CoQ10 may improve its ability in preventing oxidative stress and apoptosis resulting from mitochondrial dysfunction.

## Coenzyme Q10 and Neurodegeneration

There is increasing evidence that impairment of mitochondrial function and oxidative damage are contributing factors to the pathophysiology of Parkinson’s Disease (PD). Complex I dysfunction has been implicated in the pathogenesis of PD [13]. A recent study reported a deficit in brain CoQ10 levels in PD patients, which may be involved in the pathophysiology of PD [22]. Winkler-Stuck and co-workers [23] observed that the activity of ETC complexes, which were impaired in skin fibroblasts from a subgroup of PD patients, was restored after cultivation in the presence of 5 µM CoQ10. The neuroprotective role of CoQ10 has been also studied in other cellular models of PD, such as iron-induced apoptosis in cultured human dopaminergic neurons [24]. Iron-induced damage is mediated by ROS production and apoptosis activation; CoQ10 attenuated such iron-induced cellular damage [24]. CoQ10 has been also found to be effective in a PD mouse model of MPTP toxicity, reversing dopamine depletion, loss of tyrosine hydroxylase neurons and induction of alpha-synuclein inclusions in the substantia nigra pars compacta [23]. In PD patients, CoQ10 was well tolerated at doses as high as 1,200 mg daily (a mild effect of CoQ10 1,200 mg/day on UPDRS score has been also reported in this study) [25]. A study on 130 PD patients without motor fluctuations and a stable antiparkinsonian treatment reported that nanoparticular CoQ10 (300 mg daily) was safe and well tolerated, and led to plasma levels similar to 1,200 mg/day of standard formulations, although did not result in symptomatic effects in midstage PD [26]. The efficacy of CoQ10 in PD remains an open question [27]. A very recent short-term, randomized, placebo-controlled trial was performed in progressive supranuclear palsy (PSP). PSP, the second most common cause of parkinsonism after PD, is characterized by down gaze palsy with progressive rigidity and imbalance leading to falls. Impairment of mitochondrial ETC complex I activity has been reported in PSP [28]. CoQ10 improved cerebral energy metabolism on magnetic resonance spectroscopy studies [28]. Clinically, PSP patients improved slightly, but statistically significantly, upon CoQ10 treatment compared to placebo [28].

Huntington’s Disease (HD) is a genetic disease characterized by psychiatric disturbances, progressive cognitive impairment, choreiform movements, and death 15 to 20 years after the onset of symptoms. Various lines of evidence demonstrated the involvement of mitochondrial dysfunction in the pathogenesis of HD, but the precise role of mitochondria in the neurodegenerative cascade leading to HD is still unclear. In a mouse model of HD *in vivo* phosphorus magnetic resonance spectroscopy (31P-MRS) has been used in order to evaluate the antioxidant effect of CoQ10 and vitamin E on the activity of creatine kinase (CK), a sensitive indicator of brain energy metabolism dysfunction [29]. The results showed that CoQ10 and vitamin E prevented the increase of CK and the decrease of CoQ10 content in brain tissue, but were ineffective to prevent the decline of ETC function [29]. Smith and co-workers [30] reported that CoQ10 administration resulted to exert a therapeutic benefit in a dose dependent manner in HD mice, improving motor performance and grip strength, and reducing weight loss, brain atrophy and huntingtin inclusions [30]. Combined minocycline (an antibiotic with anti-apoptotic and neuroprotective properties) and CoQ10 therapy in a mouse model of HD ameliorated behavioral and neuropathological alterations, reduced gross brain atrophy, striatal neuron atrophy, and huntingtin aggregation, and significantly extended survival and improved motor performance to a greater degree than either minocycline or CoQ10 alone [31]. In HD patients, CoQ10 may slow the decline in total functional capacity over 30 months [32]. Kieburtz and co-workers [32] carried out a trial in which 347 patients with early HD were randomized to receive CoQ10 300 mg twice daily, remacemide hydrochloride 200 mg three times daily, both, or neither treatment, and were followed every 4 to 5 months for a total of 30 months. Patients treated with CoQ10 showed a trend toward slowing the decline in total functional capacity decline over 30 months, as well as beneficial trends in some secondary measures [32]. CoQ10 was well tolerated by HD patients.

Moreover, CoQ10 resulted to be safe in 31 subjects with Amyotrophic Lateral Sclerosis (ALS) treated with doses as high as 3,000 mg/day for 8 months [33]. ALS is a devastating disease, with selective degeneration of the anterior horn cells of the spinal cord and cortical motor neurons. The aetiology and pathogenesis of the sporadic form of the disease are poorly understood, but mitochondrial dysfunction and oxidative stress are probably involved [13]. A significant increase in the oxidized form of CoQ10 and in the ratio of oxidized form of CoQ10 to total CoQ10 have been reported in 20 sporadic ALS patients [34]. Moreover, the latter parameter significantly correlated with the duration of disease, supporting systemic oxidative stress in the pathogenesis of sporadic ALS [34]. Very recently, in order to choose between two high doses of CoQ10 for ALS and to determine if it merits testing in a Phase III clinical trial, Kaufmann and co-workers [35] performed a multicenter trial on 185 patients. There were no safety concerns, but this study showed no significant differences between CoQ10 at 2,700 mg/day for 9 months and placebo [35]. 

Most trials have demonstrated that idebenone (5 mg/kg daily) reduced cardiac hypertrophy in Friedreich’s ataxia [36]. Friedreich’s ataxia is the most common hereditary ataxia among white people, and it is caused by a trinucleotide expansion in the *X25* gene. In this disorder, the genetic abnormality results in the deficiency of frataxin, a protein targeted to the mitochondrion [36]. Although the exact physiological function of frataxin is not known, its involvement in iron–sulphur cluster biogenesis has been suggested. A possible manifestation of this disease is cardiomyopathy. A pilot study investigated the potential for high dose CoQ10/vitamin E therapy to modify clinical progression in Friedreich’s ataxia [37]. Fifty patients were randomly divided into high or low dose CoQ10/vitamin E groups [37]. At baseline serum CoQ10 and vitamin E levels were significantly decreased in patients [37]. During the trial CoQ10 and vitamin E levels significantly increased in both groups [37]. Serum CoQ10 level resulted to be the best predictor of a positive clinical response to CoQ10/vitamin E therapy [37]. Recently, a randomised, placebo-controlled trial has been conducted on 48 patients with genetically confirmed Friedreich’s ataxia [38]. Treatment with higher doses of idebenone was generally well tolerated and associated with improvement also in neurological function and activities of daily living in patients with Friedreich’s ataxia [38]. The degree of improvement correlated with the dose of idebenone, suggesting that higher doses may be necessary to have a beneficial effect on neurological function [38].

The role of mitochondrial dysfunction and oxidative stress in the pathogenesis of neurodegenerative diseases is well documented [13]. It will be important to develop a better understanding of the role of oxidative stress and mitochondrial energy metabolism in neurodegeneration, since it may lead to the development of more effective treatment strategies for these devastating disorders.

## Coenzyme Q10 Deficiency and Other Mitochondrial Disorders

There is a strong rationale for using CoQ10 supplementation to treat patients with CoQ10 deficiency [39]. CoQ10 deficiency is a rare, autosomal recessive, heterogeneous condition which has been associated with five major syndromes: (i) encephalomyopathy (with recurrent myoglobinuria, brain involvement and ragged red fibers); (ii) severe infantile multisystemic disease; (iii) cerebellar ataxia; (iv) Leigh syndrome (growth retardation, ataxia and deafness); (v) isolated myopathy [39]. Primary CoQ10 deficiencies due to mutations in ubiquinone biosynthetic genes (*i.e.*, *COQ2*, *PDSS1*, *PDSS2*, *CABC1*) have been identified in patients with the infantile multisystemic and cerebellar ataxic phenotypes [40]. In contrast, secondary CoQ10 deficiencies, due to mutations in genes not directly related to ubiquinone biosynthesis (*i.e.*, *APTX*, *ETFDH*, *BRAF*) [40], have been identified in patients with cerebellar ataxia, pure myopathy, and cardiofaciocutaneous syndrome [40]. 

The myopathic form of CoQ10 deficiency is a rare disease characterized by subacute (3–6 months) onset of exercise intolerance and proximal limb weakness without central nervous system involvement, increased serum lactate and CK levels. Frequently it is associated with lipid droplets with subtle signs of mitochondrial dysfunction at skeletal muscle level, and reduced complexes I + III and II + III activities (because CoQ10 shuttles electrons from complexes I and II to complex III of the mitochondrial ETC), and good clinical response to CoQ10 supplementation [39]. Therefore, a correct and timely diagnosis is crucial. The myopathic form of CoQ10 deficiency has been associated to mutations in the electron-transferring-flavoprotein dehydrogenase (*ETFDH*) gene [41]. *ETFDH* is also linked to another metabolic disorder, glutaric aciduria type II (GAII) [41]. Myopathic CoQ10 deficiency with pathogenic *ETFDH* mutations and late-onset GAII probably are the same disease [41]. As CoQ10 is the direct acceptor of electrons from the electron-transferring-flavoprotein, the lack of the reducing enzyme may downregulate the synthesis of CoQ10 [41]. Alternatively, faulty binding of the enzyme to CoQ10 could result in excessive degradation of the acceptor molecule [41]. Since CoQ10 deficiency/late-onset GAII is treatable, the diagnosis should be considered both in children and in adults with high-serum CK, proximal myopathy (with or without hepatopathy or encephalopathy), multiple acyl-CoA deficiency, lipid storage myopathy and decreased activity of ETC complexes I and II + III (and IV) [41]. It has been suggested that patients should be treated with both CoQ10 and riboflavin [41]. 

Infantile mitochondrial encephalomyopathy has been associated to mutations in the first and second subunits of decaprenyl diphosphate synthase (*PDSS1* and *PDSS2*), in the mevalonate pathway [40]. Mutations in *PDSS1* seem to lead to a milder phenotype than mutations in subunit 2. Patients with mutations in para-hydroxybenzoate-polyprenyl transferase (*COQ2*), a component of the CoQ10 biosynthesis complex (see Figure 2) which condenses the parahydroxybenozoate ring with the decaprenyl side-chain, share early-onset nephrosis and encephalophaty [39,40]. Very recently, a patient with primary CoQ10 deficiency whose clinical history started with neonatal lactic acidosis and who later developed multisystem disease including intractable seizures, global developmental delay, hypertrophic cardiomyopathy, and renal tubular dysfunction was reported to harbour a homozygous stop mutation affecting a highly conserved residue of *COQ9* gene, leading to the truncation of 75 amino acids [42]. Interestingly, some cases of the ataxic variant of CoQ10 deficiency have been linked to a homozygous mutation in the aprataxin (*APTX*) gene, which causes ataxia oculomotor apraxia type 1 [43]. The relationship beetween this protein, involved in DNA repair, and CoQ10 homeostasis is still unclear [43]. CoQ10 deficiency with cerebellar ataxia has been associated to mutation in *CABC1*/*COQ8*/*ADCK3* gene [44,45]. 

CoQ10 deficiency is a treatable condition, so heightened “clinical awareness” about this diagnosis is essential, especially for pediatricians and infantile neurologists. An early treatment with high-dose CoQ10 might radically change the natural history of this group of diseases [39]. Patients with all forms of CoQ10 deficiency have shown clinical improvement with oral CoQ10 supplementation, but cerebral symptoms are only partially ameliorated (probably because of irreversible structural brain damage before treatment and because of poor penetration of CoQ10 across the blood-brain barrier). Patients were given various doses of CoQ10 ranging from 90 to 2000 mg daily. The small number of patients precluded any statistical analysis but improvement was undoubtedly reported [39]. In several patients CoQ10 supplementation also ameliorated the mitochondrial function (ETC activities, lactic acid values, muscle CoQ10 content). The beneficial effects of exogenous CoQ10 require high doses and long-term administration. Also patients with ataxia oculomotor apraxia type 1 may benefit from this treatment [39].

CoQ10 deficiencies constitute a subgroup of mitochondrial disorders (MD), a group of disorders caused by impairment of the mitochondrial ETC [12]. The effects of mutations which affect the ETC may be multisystemic, with involvement of visual and auditory pathways, heart, central nervous system, and skeletal muscle [12]. The estimated prevalence of MD is 1-2 in 10000 [46]. MD are, therefore, one of the commonest inherited neuromuscular disorders. The genetic classification of MD distinguishes disorders due to defects in mtDNA from those due to defects in nDNA [12]. The first ones are inherited according to the rules of mitochondrial genetics (maternal inheritance, heteroplasmy and the threshold effect, mitotic segregation) [12]. Each cell contains multiple copies of mtDNA (polyplasmy), which in normal individuals are identical to one another (homoplasmy) [12]. Heteroplasmy refers to the coexistence of two populations of mtDNA, normal and mutated. Mutated mtDNA in a given tissue have to reach a minimum critical number before oxidative metabolism is impaired severely enough to cause dysfunction (threshold effect) [12]. Differences in mutational loads surpassing the pathogenic threshold in some tissues but not in others may contribute to the heterogeneity of phenotypes. Because of the mitotic segregation, the mutation load can change from one cell generation to the next and, with time, it can either surpass or fall below the pathogenic threshold [12]. Further, the pathogenic threshold varies from tissue to tissue according to the relative dependence of each tissue on oxidative metabolism [12]. For instance, central nervous system, skeletal muscle, heart, endocrine glands, the retina, the renal tubule and the auditory sensory cells are highly dependent on oxidative metabolism for energy generation. MD related to nDNA are caused by mutations in structural components or ancillary proteins of the ETC, by defects of the membrane lipid milieu, of CoQ10 biosynthetic genes (discussed above) and by defects in intergenomic signalling (associated to mtDNA depletion or multiple deletions) [12]. Moreover, the occurrence of a single large-scale deletion, common cause of progressive external ophthalmoplegia (PEO), is almost sporadic [12]. 

In MD patient muscle homogenates, significantly positive correlation was observed between complexes I + III and II + III activities with CoQ10 concentration [47]. CoQ10 levels resulted low in some patients with MD [48]. Recently, CoQ10 content and ETC enzyme analysis were determined in muscle biopsy specimens of 82 children with suspected mitochondrial myopathy [49]. Muscle total, oxidized, and reduced CoQ10 concentrations were significantly decreased in the probable defect group [49]. Total muscle CoQ10 was the best predictor of an ETC complex abnormality [49]. Determination of muscle CoQ10 deficiency in children with suspected MD may facilitate diagnosis and encourage earlier supplementation of this agent [49].

Therapy of MD is still inadequate, despite great progress in the molecular understanding of these disorders. Apart from symptomatic therapy, administration of metabolites and cofactors, including CoQ10, as well as of ROS scavengers, is the mainstay of real-life therapy. On the other hand, there is currently no clear evidence supporting the use of any intervention in MD [50], and further research is needed. There have been very few randomised controlled clinical trials for the treatment of MD. Those that have been performed were short, and involved fewer than 20 study participants with heterogeneous phenotypes [50].

The multitude of generally positive anecdotal data [51] together with the lack of negative side effects has contributed to the widespread use of CoQ10 in MD. In studies with eight to 44 patients CoQ10 seemed to demonstrate positive trends in mitochondrial encephalomyopathy, lactic acidosis, and stroke-like syndrome (MELAS), Kearns-Sayre syndrome (KSS), and myoclonus epilepsy with ragged red fibers (MERRF) [7]. Chen and co-workers [52] performed a randomised, double-blind cross-over trial on eight MD patients. Both subjective and objective measures showed a trend towards improvement on treatment, but the global Medical Research Council (MRC) index score of muscular strength was the only measure reaching statistical significance [52]. However, there is a need for controlled trials in large cohorts of patients [50].

Recently, Rodriguez and co-workers [53] studied the effect of a combination therapy (creatine, CoQ10, and lipoic acid) on several outcome variables using a randomized, double-blind, placebo-controlled, crossover study design in seventeen patients with various MD. Lipoic acid is found naturally within the mitochondria and is an essential cofactor for pyruvate dehydrogenase and α-keto-glutarate dehydrogenase, and is also a potent antioxidant [53]. Such combination therapy resulted in lower resting plasma lactate and a lowering of oxidative stress as reflected by a significant reduction in urinary 8-isoprostanes and a directional trend in 8-hydroxy-2’-deoxyguanosine (8-OHdG) excretion [53]. Isoprostanes are prostaglandin-like compounds formed by the peroxidation of arachadonic acid, and are considered one of the most reliable markers to assess oxidative stress *in vivo*. 8-OHdG is formed by the hydroxylation of guanosine residues and is often used as a biomarker of oxidative damage to DNA. Further, the combination therapy attenuated the decrease in peak ankle dorsiflexion strength that was observed following the placebo phase [53]. 

A synthetic shorter chain CoQ10 analogue is idebenone. It has been reported to improve brain and skeletal muscle metabolism in isolated cases of MD, and seemed to enhance the rate and degree of visual recovery in Leber Hereditary Optic Neuropathy [50]. 

Therapy for MD remains inadequate and mostly symptomatic, but the rapidly increasing knowledge of their molecular defects and pathogenic mechanisms allows for some cautious optimism about the development of effective treatments in the next future [12]. One of the “cocktails” of choice for the treatment of MD may be a combination of L-carnitine (1,000 mg three times a day) and CoQ10 (at least 300 mg a day), with the rationale of restoring free carnitine levels and exploiting the oxygen radical scavenger properties of CoQ10 [12]. 

## Statin Myopathy

Statins are currently the most effective medications for reducing low-density lipoprotein (LDL) cholesterol concentrations [54]. Statins competitively inhibit HMG-CoA reductase thereby decreasing synthesis of mevalonate, a critical intermediary in the cholesterol synthesis pathway [54]. Their most serious and frequent side effects are a variety of myopathic complaints ranging from mild myalgia to fatal rhabdomyolysis [54]. Statins block production of farnesyl pyrophosphate, an intermediate in the synthesis of CoQ10 [54]. 

The fact that statins block the mevalonate pathway has prompted the idea that statin-induced CoQ10 deficiency may be involved in the pathogenesis of statin myopathy (the primary adverse effect limiting their use) [54]. Therefore, supplementing with CoQ10 may be recommended to prevent the myopathic side effects associated with the statins. Evidences for or against this hypothesis have been reviewed by Marcoff and Thompson [54], but the question remains to be answered. A study performed on a sample of muscle biopsy of patients with statin drug-related myopathy showed that the decrease of CoQ10 concentration in muscle did not cause histochemical or biochemical evidence of mitochondrial myopathy or morphologic evidence of apoptosis in most patients [55]. 

A study designed to assess the effect of high-dose statin treatment has been performed on 48 patients with hypercholesterolemia, randomly assigned to receive simvastatin, atorvastatin, or placebo for 8 weeks [56]. Muscle ubiquinone concentration was reduced significantly in the simvastatin group, but no reduction was observed in the atorvastatin or placebo group [56]. Also ETC and citrate synthase activities were reduced in patients taking simvastatin [56]. 

The effect of simvastatin on CoQ10 plasmatic levels has been compared with the effect of ezetimibe (a cholesterol absorption inhibitor) and of the coadministration simvastatin/ezetimibe [57]. While simvastatin and the combination of simvastatin and ezetimibe significantly decreased plasma CoQ10 levels, ezetimibe monotherapy did not [57].

A randomized double-blind, placebo-controlled study that examined the effects of CoQ10 and placebo in hypercholesterolemic patients treated by atorvastatin showed a similar decrease in LDL carriers in the two groups and an high increase of CoQ10 levels in the CoQ10 group [58]. The placebo group showed a mean reductions of plasma CoQ10 levels by 42%, whereas patients supplemented with CoQ10 showed a mean increase in plasma CoQ10 by 127% [58]. However, these changes in plasma CoQ10 levels showed no relation to the changes in serum transaminase and CK levels [58]. Further studies are needed in order to evaluate the role of CoQ10 supplementation during statin therapy [54].

## Migraine

Sandor and co-workers [3] compared CoQ10 (3 × 100 mg/day) and placebo in 42 migraine patients in a double-blind, randomized, placebo-controlled trial. CoQ10 was superior to placebo for attack-frequency, headache-days and days-with-nausea in the third treatment month [3]. Therefore, it has been suggested that CoQ10 was efficacious and well tolerated.

More recently, Hershey and co-workers [4] measured CoQ10 levels in 1550 patients with paediatric and adolescent migraine headache, and found that 32.9% were below the reference range. Patients with low CoQ10 were recommended to start 1 to 3 mg/kg per day of CoQ10 in liquid gel capsule formulation. In a subset of patients who returned for timely follow-up total CoQ10 levels improved (*P* < 0.0001) and the headache frequency and disability seemed to reduce (*P* < 0.001). More rigorous studies are needed in order to evaluate if determination of CoQ10 levels and consequent supplementation may result in clinical improvement in migraine patients [4].

## Conclusions

The generalized mitochondrial defect might be *in vitro* ameliorated by CoQ10 treatment [15]. In addition to primary CoQ10 deficiency, CoQ10 treatment may have some efficacy in the treatment of MD and neurological disorders not directly linked to a primary deficiency in this quinone, but in general terms linked to mitochondrial dysfunction and oxidative stress [13]. CoQ10 therapy has been shown to be relatively safe as far as the adverse effects. Further studies on the potential usefulness of CoQ10 supplementation in neurological diseases are strongly needed, in case also with water-soluble formulations, which have been suggested to improve the ability of CoQ10 in preventing oxidative stress and apoptosis resulting from mitochondrial dysfunction [21].
